# Genetic factors associated with cancer racial disparity – an integrative study across twenty‐one cancer types

**DOI:** 10.1002/1878-0261.12799

**Published:** 2020-09-24

**Authors:** Yan Li, Xiaodong Pang, Zihan Cui, Yidong Zhou, Feng Mao, Yan Lin, Xiaohui Zhang, Songjie Shen, Peixin Zhu, Tingting Zhao, Qiang Sun, Jinfeng Zhang

**Affiliations:** ^1^ Department of Breast Surgery Peking Union Medical College Hospital Peking Union Medical College Chinese Academy of Medical Sciences Beijing China; ^2^ Insilicom LLC Tallahassee FL USA; ^3^ Department of Statistics Florida State University Tallahassee FL USA; ^4^ Boston Biosciences Inc. Boston MA USA; ^5^ Broad Institute of Harvard & MIT Cambridge MA USA; ^6^ McGovern Institute for Brain Research MIT Cambridge MA USA; ^7^ Department of Geography Florida State University Tallahassee FL USA

**Keywords:** cancer racial disparity, genetic drivers, immunotherapy, integrative genomics, single nucleotide polymorphisms, XKR9

## Abstract

It is well known that different racial groups have significantly different incidence and mortality rates for certain cancers. It has been suggested that biological factors play a major role in these cancer racial disparities. Previous studies on the biological factors contributing to cancer racial disparity have generated a very large number of candidate factors, although there is modest agreement among the results of the different studies. Here, we performed an integrative analysis using genomic data of 21 cancer types from TCGA, GTEx, and the 1000 Genomes Project to identify biological factors contributing to racial disparity in cancer. We also built a companion website with additional results for cancer researchers to freely mine. Our study identified genes, gene families, and pathways displaying similar differential expression patterns between different racial groups across multiple cancer types. Among them, XKR9 gene expression was found to be significantly associated with overall survival for all cancers combined as well as for several individual cancers. Our results point to the interesting hypothesis that XKR9 could be a novel drug target for cancer immunotherapy. Bayesian network modeling showed that XKR9 is linked to important cancer‐related genes, including FOXM1, cyclin B1, and RB1CC1 (RB1 regulator). In addition, metabolic pathways, neural signaling pathways, and several cancer‐related gene families were found to be significantly associated with cancer racial disparities for multiple cancer types. Single nucleotide polymorphisms (SNPs) discovered through integrating data from the TCGA, GTEx, and 1000 Genomes databases provide biologists the opportunity to test highly promising, targeted hypotheses to gain a deeper understanding of the genetic drivers of cancer racial disparity and cancer biology in general.

## Graphical abstract

Based on an integrative analysis using genomic data of 21 cancer types from TCGA, we identified biological factors contributing to racial disparity in cancer: XKR9 gene expression is significantly associated with overall survival for all cancers combined as well as for several individual cancers, suggesting that XKR9 could be a novel drug target for cancer immunotherapy.

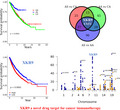

**Abbreviations**
AA: African Americans
API: Asian/Pacific Islander
AS: Asian Americans
BLCA: bladder cancer
BN: Bayesian network
BRCA: breast cancer
CA: Caucasian Americans
CXCLs: C‐X‐C motif chemokine ligands
DE: differentially expressed
DEGs: differentially expressed genes
DGEA: differential gene expression analysis
FOXM1: forkhead box M1
KICH: kidney chromophobe
KIRP: kidney renal papillary cell carcinoma
KLKs: kallikreins
LGG: low‐grade glioma
LIHC: liver hepatocellular carcinoma
MAGEs: melanoma antigen gene
MTs: metallothioneins
MUCs: mucins
NHB: non‐Hispanic black
NHW: non‐Hispanic white
RB1CC1: RB1 regulator
SNPs: single nucleotide polymorphisms;

## 1 Introduction

Cancer is the second most common cause of death in the United States after heart disease [1]. It has a greater economic impact from premature death and disability than all causes of death combined worldwide [2]. It is well known that different racial groups have significantly different incidence and mortality rates for certain cancers [3, 4, 5, 6, 7, 8, 9]. For all cancer types combined, the incidence rates in the United States are 465.3, 463.9, and 291.7 per 100 000 people, for non‐Hispanic white (NHW), non‐Hispanic black (NHB), and Asian/Pacific Islander (API) populations, respectively [1]. The mortality rates for all cancers combined are 165.4, 190.6, and 100.4 per 100 000 people for NHW, NHB, and API populations, respectively [1]. NHWs and NHBs have the highest incidence rates; NHBs have the highest mortality rates, followed by NHWs, and APIs have much lower incidence and mortality rates than both NHWs and NHBs.

The causes of cancer racial disparities have been studied extensively [10, 11, 12, 13, 14, 15, 16, 17, 18, 19], and socioeconomic status has been found to be an independent predictor of mortality [12, 13, 14]. However, even when socioeconomic status has been accounted for, there are still substantial disparities that remain among different races [20, 21]. It has been suggested that biological factors play a major role in these cancer racial disparities [22, 23, 24, 25, 26]. There is a large volume of literature studying the biological factors contributing to racial disparities among different races for various cancers [11, 16, 17, 18, 19, 39]. In Table S1, we list a subset of the genetic factors previously described as associated with racial disparity in breast cancer and prostate cancer, as they have been the most frequently studied cancers. In addition to genetic factors, epigenetic factors have also been investigated in a number of previous studies [27]. For prostate cancer, several studies have found consistent regions containing SNPs highly associated with prostate cancer risk [37, 38, 39, 40], potentially explaining some of the racial disparity observed between African and Caucasian men. However, few of the identified prostate cancer risk variants align to known genes or appear to alter the coding sequence of an encoded protein [39].

Previous studies on the biological factors contributing to cancer racial disparity have generated a very large number (usually at the level of hundreds) of candidate factors, both genetic and epigenetic, with modest agreement among the results of these studies. This is likely due to the limited sample size in some of the previous studies as well as the differences in the populations from which the samples were selected. Each of the previous studies has addressed individual cancer types, and studies on different cancers have identified quite varied genetic and epigenetic factors. Furthermore, few studies have been conducted to investigate less common cancers.

**Fig. 1:**
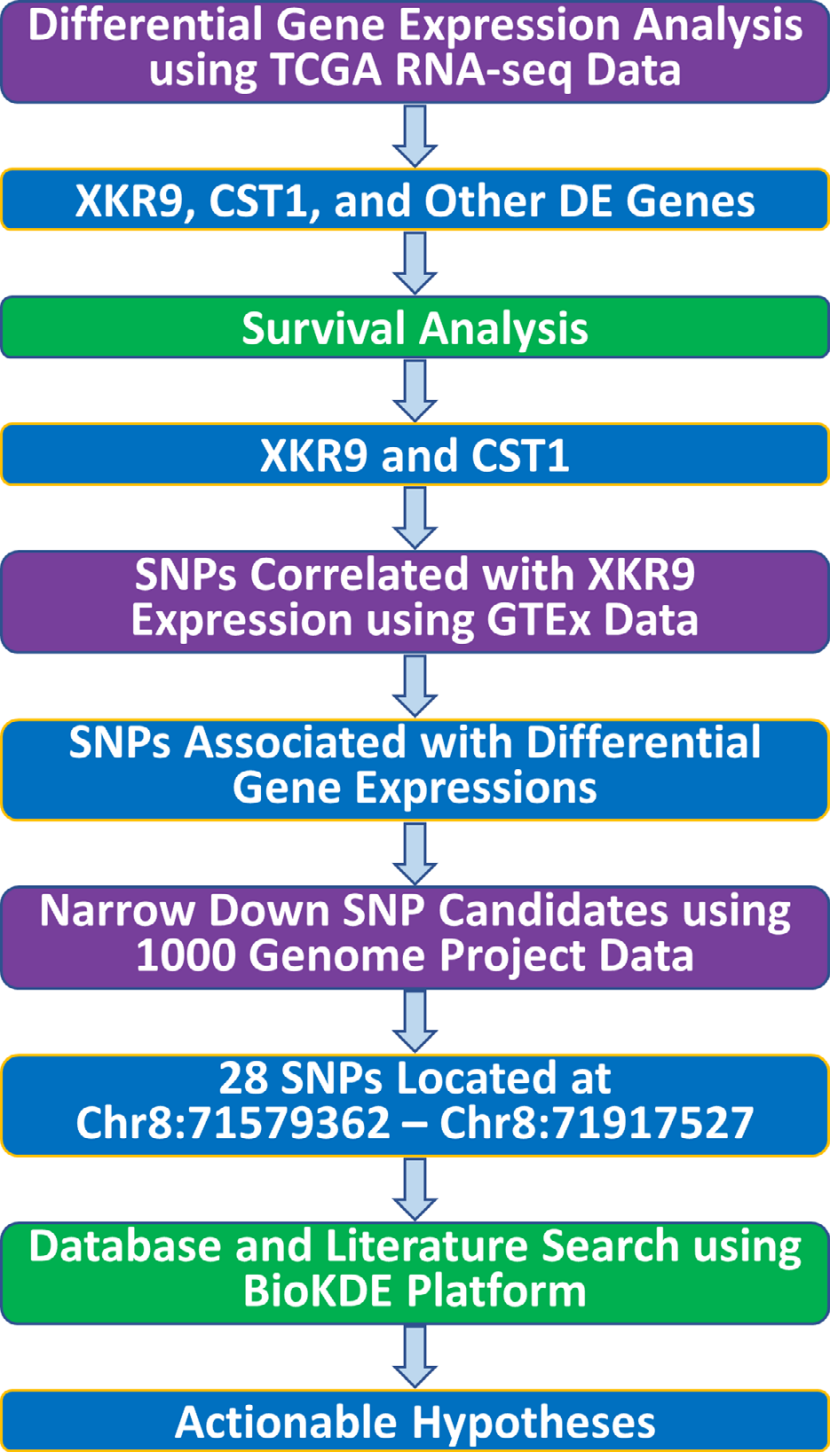
The overview of our data analysis pipeline. The following data sources are used: TCGA RNA‐seq data with race information, GTEx SNP and gene expression correlations, 1000 Genomes Project SNP genotype percentages in different races, GeneCards database for gene‐related information, and BioKDE (https://biokde.com) for literature search.

Although the gene expression differences between tumors of different racial groups have been well established by previous studies in multiple cancer types, the drivers behind the gene expression differences have been poorly understood. To identify the potential genetic drivers of cancer racial disparity, we applied an integrative analysis approach using multiple datasets as illustrated in Fig. 1. We started from gene expression data (RNA‐seq) in TCGA by conducting differential gene expression analysis (DGEA) comparing gene expressions of tumor samples from patients of different races. This generated differentially expressed (DE) genes between two specific groups which are defined by the races of the groups and the cancer type (or all cancer combined). Since some of the DE genes may be associated with race only, but not cancer, survival analysis using the DE genes was conducted to find DE genes that are significantly associated with overall survival of the cancer patients (XKR9 and CST1 were found in this study). To identify the driver genetic factors, we then used GTEx data to find SNPs correlated significantly with the expression of XKR9 or CST1. To ensure the SNPs are able to explain the KXR9 or CST1 differential expression, the consistency between differential gene expression with the proportions of the alternative allele in different race groups (using 1000 Genomes Project data) and the effect of the alternative allele to gene expression (using GTEx data) are checked, which further narrowed the list of SNPs down to a much smaller set of SNPs. Database (UCSC genome browser, GeneCards, etc.) searches were performed to collect more information of the genes and SNPs. The final step is literature search using BioKDE platform (https://biokde.com) to validate the discoveries and generate specific hypotheses which can be tested experimentally.

Differential gene expression analysis using all cancer types allowed us to substantially increase the sample size to effectively eliminate the number of false positives in identified differentially expressed genes (DEGs). Pathway analyses identified cancer‐related pathways, which allow us to focus on genes and pathways that are truly associated with cancer racial disparities, instead of being associated with race only. We performed analyses using data with all cancers combined and data from individual cancer types. The majority of our analyses are focused on results taken from all cancers combined, and the companion website (https://rep.insilicom.com/cancer_racial_disparity/index.html) contains details of all the results.

Our study is the first such large‐scale study on the genetic factors of cancer racial disparity. It provides a comprehensive picture of the genetic factors associated with cancer racial disparity across multiple cancers. Furthermore, SNPs identified from the integrative analysis point to potential genetic drivers, which can be tested in future validation studies.

## 2 Materials and Methods

The data used in this study are described in Supporting Information.

### 2.1 Statistical analysis

### 2.2 Differential gene expression analysis

For the overall comparison with all cancers combined, Limma package [41] was used to perform three pairwise comparisons: Asian Americans (AS) vs. Caucasian Americans (CA), AS vs. African Americans (AA), and AA vs. CA. For the DGEA of individual cancer types, DESeq2 package [42] was used.

### 2.3 Pathway analysis

The pathway analysis was performed using QIAGEN’s Ingenuity Pathway Analysis (IPA) software (https://www.qiagen.com/).

### 2.4 Survival analysis

Survival analysis was performed using survival (Therneau T (2020). *A Package for Survival Analysis in R*. R package version 3.1‐12, https://CRAN.R‐project.org/package=survival) and survminer (Alboukadel Kassambara (2020). *survminer: Survival Analysis and Visualization*. R package version 0.4.7, https://CRAN.R‐project.org/package=survminer) packages in R.

### 2.5 Heatmap and clustering analysis

We used Ward’s method [43] for hierarchical clustering and Euclidean distance for clustering rows and columns. R package pheatmap (Perry M (2020). *heatmaps: Flexible Heatmaps for Functional Genomics and Sequence Features*. R package version 1.12.0) was used for plotting the heatmap.

### 2.6 Association of SNPs with gene expressions

Combining data obtained from GTEx eQTL studies, the 1000 Genomes Project, and TCGA, we infer specific SNPs that may be associated with the differential expression patterns observed from the differential gene expression analysis of TCGA gene expression data following a previously developed protocol [11, 29]. Specifically, we searched for SNPs that may be associated with the initially identified DEGs. The conditions used for selecting SNPs required that the following should all be consistent with one another: (1) the allele frequencies of the SNPs in different races (1000 Genomes Project), (2) the association between the genotype of the SNPs with differential expression patterns (GTEx), and (3) the differential expression patterns in different races (TCGA). For example, from the TCGA data we found that XKR9 is down‐regulated in AS patient samples compared to AA and CA samples, and its expression is lower in AA samples compared to CA samples (Table 1). From the GTEx, we identified a SNP, rs17689585, which is associated with XKR9 expression, and the effect of this common allele is increased expression of the XKR9 gene. The frequencies of the most common allele are 0.0744, 0.2247, and 0.5149 for AS, AA, and CA racial groups, respectively. This SNP can potentially explain the differential expression of XKR9 across the three races. Manhattan plot showing the SNPs was generated using R package qqman (Turner, S.D. qqman: an R package for visualizing GWAS results using Q‐Q and manhattan plots. biorXiv https://doi.org/10.1101/005165).

**Table 1:** Selected differentially expressed genes and transcripts across three races for all cancer types. The genes are among the top differentially expressed genes, and literature search showed some interesting findings about them. The numbers in columns 2, 3, and 4 are the log2 fold change and p‐values (in parentheses). AS vs. CA: Asian American cancer samples compared to Caucasian American cancer samples, with CA samples used as the reference set. AS vs. AA: Asian American samples compared to African American samples, with AA samples used as the reference. AA vs. CA: African American samples compared to Caucasian American samples, with CA used as the reference. NDE: nondifferentially expressed

Gene	AS vs. CA	AS vs. AA	AA vs. CA	Functions
XKR9	−1.6 (3.3e‐43)	−1.1 (8.1e‐11)	−0.63 (4.1e‐16)	Exposing phosphatidylserine during apoptosis
CST1	0.69 (4.27e‐04)	1.67 (1.20e‐07)	−0.76 (3.17e‐07)	Regulation of cell proliferation, clone formation, and metastasis
SIGLEC14	−1.5 (2.2e‐44)	−0.97 (3.9e‐8)	NDE	Regulation of immune cell functions. It activates immune cells by recruiting Syk.
SIGLEC12	1.2 (9.7e‐24)	1.2 (1.2e‐11)	NDE	Regulation of immune cell functions. It may protect against the development of SLE in Asian populations.
UGT2B17	−1.4 (1.2e‐14)	−1.6 (1.3e‐7)	NDE	Conjugation and subsequent elimination of potentially toxic xenobiotics and endogenous compounds
CHIT1	−1.2 (2.0e‐13)	−1.15 (3.0e‐6)	NDE	Known SNPs associated with colorectal cancer
MTRNR2L1	−1 (7.9e‐10)	NDE	−1.3 (1.5e‐24)	Cell life and antiapoptosis

### 2.7 Bayesian network modeling

We used a biological network inference method, GRASP, which we recently developed [44] to build a Bayesian network (BN) model for selected genomic features using several types of genomic data available from TCGA, including mRNA‐seq, microRNA‐seq, protein expression, and DNA methylation data. We first used RNA‐seq data to identify transcripts highly correlated with a feature of interest (XKR9 gene expression in this study). There are hundreds of genes with significant correlation coefficients with XKR9 as measured by adjusted p‐values (padj ≤ 0.05). We chose relatively large correlation coefficient cutoffs to give us a small number of variables to control the size of the resulting network. Six transcripts (mRNA‐seq) were selected whose absolute values of correlation coefficients with that of XKR9 are greater than 0.38. We then included other genomic features, including microRNAs, DNA methylations, and protein expressions that are highly correlated with these transcripts, which produced 10 microRNAs (absolute correlation coefficient > 0.45), 10 DNA methylation regions aggregated around genes (absolute correlation coefficient > 0.4), and 3 protein expressions (absolute correlation coefficient > 0.3). The details of the BN structure learning method are given in Ref. [44].

The companion website, https://rep.insilicom.com/cancer_racial_disparity/index.html, provides more details on the data and methods we used in this study.

## 3 Results

**Fig. 2:**
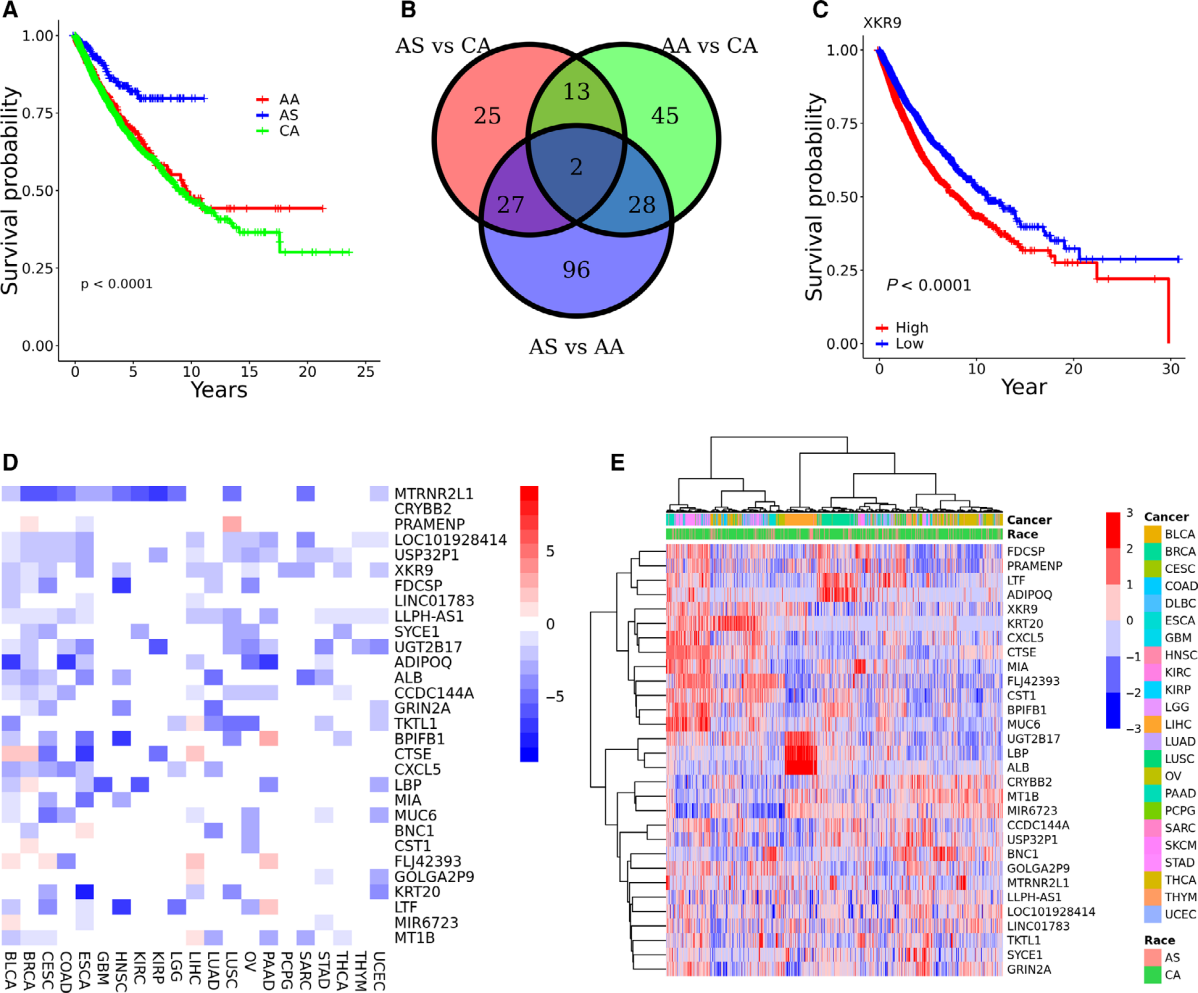
Results for all cancer types combined. (A) Survival curve for the three races; (B) Venn diagram of DEGs among three comparisons; (C) survival plots of XKR9. The median value of XKR9 expression was used to separate the population into high and low groups; (D,E) heatmaps of the selected top 30 differentially expressed genes between AS and CA tumors, where the color schemes show log2 fold change in D and log2 of normalizing the expression of genes at target library of 40 million in E, respectively.

In this study, we performed integrative genomics analysis across three races: Asian American (AS), Caucasian American (CA), and African American (AA) for all 21 cancer types combined as well as each individual cancer type. The patients are races matched with AS:AA:CA of 1:1:4. There are totally 289 AS, 289 AA, and 1156 CA patients. From TCGA data, racial disparity in overall survival is evident when pooling patients from all the cancer types: AS has much higher survival probability than CA and AA, while CA and AA have comparable survival probability to each other (Fig. 2A). For most of the analyses, we present the results from the comparison of AS vs CA in the main text and provide results of the other two pairwise comparisons in the Supporting Information or on the companion website.

### 3.1 Differential gene expression analysis across three races for all cancer types

We first looked at differentially expressed genes using combined RNA‐seq data from all cancer types. Three pairwise comparisons were performed for AS vs. CA, AS vs. AA, and AA vs. CA. As shown in Fig. 2B, for the AS vs. CA comparison, using a fold change of 1.5 (|log2 fold change|≥0.585) and an adjusted p‐value <= 0.05, we identified a total of 67 differentially expressed genes (DEGs), including 42 down‐regulated and 25 up‐regulated genes, with CA used as the reference group. For the AA vs. CA comparison, using the same criteria, we obtained a total of 88 DEGs, including 42 down‐regulated and 46 up‐regulated genes, again with CA used as the reference group. For the AS vs. AA comparison, we identified 153 DEGs, including 108 down‐regulated DEGs and 45 up‐regulated DEGs, this time using AA as the reference group. Two genes (XKR9 and CST1) are found to be DEGs among all the three comparisons (Fig. 2B). A survival analysis was performed on the DEGs, and XKR9 was found to have a significant effect on survival when considering all of the cancer types combined (Fig. 2C) as well as in three specific cancer types (kidney renal papillary cell carcinoma (KIRP), kidney chromophobe (KICH), and liver hepatocellular carcinoma (LIHC)) (Figs S1A, S1B, and S1C). Reduced expression of XKR9 is always associated with higher survival probabilities. XKR9 was found to be down‐regulated in AS cancer patients in both the AS vs. CA comparison (log2 fold change = −1.58; adjusted p‐value = 1.50e‐39) and the AS vs. AA comparison (log2 fold change = −1.08; adjusted p‐value = 6.01e‐08). CST1 was found to have different effect on survival in three individual cancer types with elevated express of CST1 associated with higher survival probability in BLCA and BRCA (the higher the expression of CST1, the higher the overall survival time of the patients), while reduced expression of CST1 associated with higher survival probability in LGG (the lower the expression of CST1, the higher the overall survival of the patients) (Figs S1D, S1E, and S1F). Several other genes known to be involved in cancer were also found to be differentially expressed (Table 1).

We then looked at the differentially expressed genes in individual cancer type and identified top 30 DEGs between AS and CA tumors in individual cancer types as shown in Fig. 2D,E based on a total of 63 comparisons (21 cancer types and three comparisons in each, see below for more details). We can see that most of the DEGs are down‐regulated in AS tumors, which is consistent with what we have observed in previous studies [11, 29]. The correlation matrix of the genes in Fig. 2D was calculated and plotted to provide an overview on the correlation of these DEGs (Fig. S3 in Supplementary Materials). Results of the other two pairwise comparisons are given in Supplementary Materials (Fig. S2).

### 3.2 Pathway and upstream regulator analysis

**Fig. 3:**
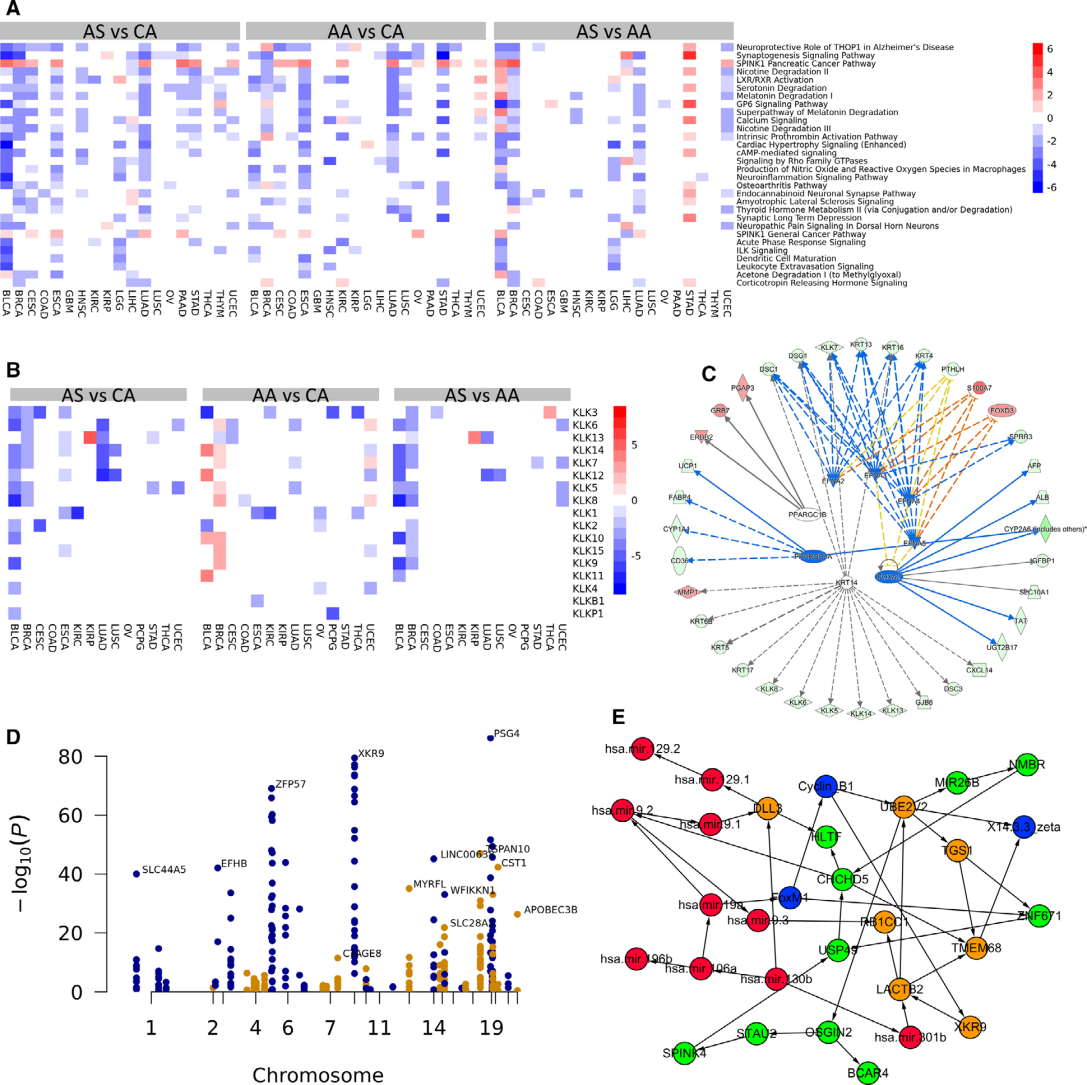
(A) Heatmap of z‐score of dysregulated pathways for three pairwise cancer‐type‐specific comparisons; (B) heatmap of KLK genes for three pairwise cancer‐type‐specific comparisons; (C) upstream regulators and the genes they regulate in breast cancer racial disparity study; (D) Manhattan plot showing the SNPs identified for the AS vs CA comparison using data for all cancer types combined. The different colors are used to help distinguish SNPs in different regions/chromosomes; (E) the network learned by employing a Bayesian network model that uses RNA‐seq, protein expression, DNA methylation, and microRNA‐seq data to understand the potential function of XKR9. Orange circles: nodes representing mRNA expression of genes; Red circles: nodes representing microRNA; Blue circles: nodes representing protein expressions; Green circles: DNA methylations.

A pathway analysis using the Ingenuity Pathway Analysis (IPA) suite using differentially expressed genes from overall comparison showed that a number of pathways are dysregulated in multiple cancers in the AS vs. CA comparison (Fig. 3A) as well as in the other two comparisons. The majority of the pathways are down‐regulated in AS tumors except for the SPINK1 pancreatic cancer pathway and the SPINK1 general cancer pathway. Among the dysregulated pathways are several metabolism‐related pathways such as nicotine degradation II, LXR/RXR activation, serotonin degradation, melatonin degradation I, the superpathway of melatonin degradation, nicotine degradation III, thyroid hormone metabolism, and acetone degradation I. Several neural pathways were also found to be significantly dysregulated, including those known as the neuroprotective role of THOP1 in Alzheimer’s disease, synaptogenesis signaling pathway, neuroinflammation signaling pathway, endocannabinoid neuronal synapse pathway, synaptic long‐term depression, and neuropathic pain signaling in Dorsal horn neurons.

### 3.3 Differential gene expression analysis for individual cancer types

To identify DEGs that may be only specific to certain individual cancers, we performed a DGEA for individual cancers. Surprisingly, unlike the overall comparison with all cancer types, the comparisons of individual cancers generally produced a much larger number of DEGs with larger‐fold changes and smaller p‐values, despite utilizing much smaller sample sizes. To further focus the results of our analysis, we manually checked the DEGs and selected a small number of gene families whose members have similar differential expression patterns in more than one type of cancer. These gene families are more likely to be truly differentially expressed given these two conditions: (1) more than one member of these gene families have similar differential expression patterns in a particular comparison in one cancer type; and (2) members of these families have similar differential expression patterns in more than one cancer type. The gene families selected are kallikreins (KLKs), metallothioneins (MTs), mucins (MUCs), melanoma antigen gene (MAGEs), and C‐X‐C motif chemokine ligands (CXCLs). Figure 3B shows the heatmap of the KLK gene family for cancers that include at least one KLK gene detected as a DEG.

More discussions of this study are provided in Supporting Information.

### 3.4 Genetic factors associated with breast cancer racial disparity—a case study

In addition to the analysis of the combined data from all 21 cancer types, we performed similar analyses on individual cancers and made all of the results available on the companion website. Each cancer type has a separate webpage documenting all of the results related to that cancer type. Researchers are encouraged to explore the website to mine new discoveries on cancer racial disparities specific to individual cancers. Here, we use breast cancer as an example illustrating how the individual cancer results can be explored. For breast cancer, there are a total of 781 CA, 184 AA, and 61 AS patients. The differential gene expression analysis returned 1319, 1309, and 1458 DEGs, for the AS vs. CA, AA vs. CA, and AS vs. AA comparisons, respectively, using a fold change of 2.0 (|log2 fold change|>= 1.0) and an adjusted p‐value <= 0.05. Clicking on gene names in the table will display the box plot and histogram for the expression of the genes across the three racial groups. This can be quite informative as some DEGs are false positives driven by some outlier genes, which could not be effectively detected by the DESeq2 program. Visual inspection will identify such genes. The ‘NCBI’ and ‘GeneCards’ links can also take users to the corresponding webpage of the genes at NCBI or the GeneCards database. The Venn diagram shows the overlap among the three comparisons, the heatmap shows the expression of DEGs, and the survival analysis plot shows the survival curves for patients across the three racial groups. In addition, we also performed a pathway analysis using the IPA suite, which includes dysregulated pathways, upstream regulators, and diseases/functions related to the DEGs. A graph containing upstream regulators and the genes they regulate is shown in Fig. 3C.

### 3.5 SNPs associated with differentially expressed genes

A fundamental question of disease studies is to understand how genotypes affect the phenotypes of the disease of interest. It has been well established that common genetic variants account for differences in gene expression among ethnic groups [45]. In this study, we focused on two phenotypes of interest: the outcome disparity among CA, AA, and AS cancer patients and the genome‐wide expression profiles of all the transcripts from tissue samples of twenty‐one cancers. To identify genotypes associated with these phenotypes, we combined gene expression data with eQTL data from the GTEx project and SNP allele frequency information from the 1,000 Genomes Project [11, 29] (see Materials and Methods for details). Figure 3D displays the SNPs identified from the AS vs. CA comparison using a Manhattan plot. We can see that many SNPs are clustered around certain regions, indicating they are likely to be truly associated with cancer racial disparity. The determination of whether or not they are driver SNPs would require further experimental studies.

### 3.6 Understanding the mechanism of the association between XKR9 and cancer health disparity using Bayesian network models

XKR9 was found to be differentially expressed in all three pairwise comparisons of combined data for all cancers. XKR9 expression was also found to be associated with overall survival. Several SNPs associated with the differential expression of XKR9 were discovered using an integrative analysis combining data from the TCGA, GTEx, and 1000 Genomes Project databases. The allele frequencies in different races are also consistent with their differential expression patterns, indicating the SNPs are potential genetic drivers for cancer racial disparities across multiple cancer types. A literature search for XKR9 returned little information on its function and its relationship with cancer. Using mRNA‐seq, microRNA‐seq, protein expression, and DNA methylation data obtained from TCGA, we inferred a Bayesian network (BN) structure (Fig. 3E) for the selected genomic features using our BN structure learning method, GRASP, described in Method. The learned network suggests that XKR9 is regulated by a regulatory protein, cyclin B1, which was regulated by forkhead box M1 (FOXM1). Regulation of cyclin B1 by FOXM1 is well established in literature [46, 47]. FOXM1 (forkhead box protein M1) is an important proliferation‐associated transcription factor widely spatiotemporally expressed during cell cycle. It is involved in the processes of cell proliferation, self‐renewal, and tumorigenesis [48]. Due to its potential role as molecular target in cancer therapy, FOXM1 was named the Molecule of the Year in 2010 [49, 50]. In the BN, XKR9 directly regulates LACTB2, which regulates other genes and microRNAs including a tumor suppressor gene, RB1CC1. RB1CC1 is a potential regulator of the tumor suppressor, RB1. From both upstream and downstream proteins/genes identified by the BN, XKR9 likely plays some intriguing functions in cancer. The BN model links XKR9 expression with tumor growth and apoptosis, which may explain its effect on overall survival of cancer patients. The model also provides guidance for formulating well‐defined hypotheses for future validation studies.

## 4 Discussion

Despite extensive previous studies on the topic, a substantial proportion of cancer racial disparities still cannot be explained by known factors. Many previous studies have identified biological factors associated with the disparities. However, the agreement among these studies is rather poor. Limited sample sizes, sampling population differences, and/or randomness in multiple comparisons may have all contributed to the discrepancy. Most previous studies have focused on individual cancers and identified quite different genetic factors for different cancer types. A natural question one may ask is as follows: Are these racial disparities in different cancer types isolated events? It is well known that cancers from different organs can share very similar characteristics if they have similar driver mutations. The genetic factors affecting cancer racial disparities likely act across multiple cancers as well. Another question is as follows: With so many candidate genetic factors discovered in the past, which ones are the drivers? Identification of driver genetic factors that cause cancer racial disparity would help researchers design effective approaches to reduce or eliminate the disparities.

In this study, we used TCGA genomic data from a total of 7,789 patient samples representing twenty‐one cancer types. Our study discovered a number of genes which are differentially expressed in multiple cancers between different racial groups. These genes are more likely to be truly associated with cancer racial disparity compared to the genes identified using smaller sample sizes from studies examining individual cancer types. One of the DEGs, XKR9, was found to be associated with overall survival for all of the cancers combined as well as for three individual cancer types. We further identified pathways differentially regulated across multiple cancer types. An additional differential expression analysis using individual cancers identified more DEGs, gene families, and pathways in individual cancer types. Combining data from three major public databases—TCGA, GTEx, and 1000 Genomes—allowed us to find promising candidate SNPs, which could be the genetic drivers of cancer racial disparity. We have put all of the results on a companion website available at https://rep.insilicom.com/cancer_racial_disparity/index.html. There is still much to be discovered from our analysis results, and we welcome cancer researchers to utilize these resources to make new discoveries. Below, we discuss some of our discoveries in more detail.

We used UCSC genome browser to find the location of the SNP, rs17689585, which was found to be associated with XKR9 gene expression and may be a potential driver SNP for cancer racial disparity (Fig. S4). The SNP overlaps with gene, AC015687.1, which is a novel transcript. Searching AC015687.1 at GeneCards database (https://www.genecards.org/cgi‐bin/carddisp.pl?gene=ENSG00000285579) showed that this gene is an enhancer for XKR9. A few other important transcription factors bind this region of the genome, including FOXA2, RXRA, HNF4A, YY1, ZNF644, FOXA1, SP1, SOX13, and HNF4G. Among them, HNF4A, HNF4G, and FOXA2 correlate relatively strongly to XKR9, indicating they may play regulatory roles on XKR9 (Fig. S5). These discoveries point to some actionable hypotheses, which can be tested experimentally. For example, site‐directed mutagenesis could be done to see whether the SNPs in this region indeed affect XKR9 expression and how they affect the physiology of cells. One can also check whether the mutations regulate XKR9 expression through modifying the binding of any of the transcription factors.

Given that XKR9 (XK related 9) could be the gene directly regulated by one or more of the transcription factors (FOXA2, RXRA, HNF4A, YY1, ZNF644, FOXA1, SP1, SOX13, and HNF4G) through one or more of the SNPs (multiple SNPs around chr8:71579362 and chr8:71917527) to cause some of the observed cancer racial disparities, we conducted literature search to find more about the links between XKR9 and cancer. It has been shown that apoptotic cells expose phosphatidylserine (PtdSer) on their surface as an ‘eat me’ signal and mammalian Xk‐related (Xkr) proteins promote apoptotic PtdSer exposure [51, 52]. A recent study found that this pathway can be hijacked by tumor cells to suppress the development of immunity toward tumor cells, which implies that PtdSer blockade by the therapeutic administration of PS‐targeting agents may restore pathogen and tumor immunity [53]. Our study indicates another opportunity by targeting on XKR9 to regulate the exposure of PtdSer on cell surface. The observation that higher expression of XKR9 is associated with lower survival probability also suggests that targeting on XKR9 may be worth exploring as a potentially new therapeutic direction in cancer immunotherapy.

## 5 Conclusions

In this study, we found that higher expression of CST1 is positively correlated with longer overall survival for breast cancer (BRCA) and bladder cancer (BLCA), while it is negatively correlated with overall survival for low‐grade glioma (LGG). This is in contradiction with a previous study indicating that elevated CST1 expression promotes breast cancer progression and predicts a poor prognosis [54]. Literature review showed that CST1 may play different roles in different cancers [55, 56, 57, 58], but failed to reconcile the discrepancy between previous studies and ours. Further experimental work is needed to shed more light on the role CST1 plays in cancer.

Our study identified several neural pathways differentially regulated across multiple cancer types. These include the pathways known as the neuroprotective role of THOP1 in Alzheimer’s disease, synaptogenesis signaling pathway, neuroinflammation signaling pathway, endocannabinoid neuronal synapse pathway, synaptic long‐term depression, and neuropathic pain signaling in Dorsal horn neurons. Recent studies have found that the central nervous system and sympathetic nervous systems may play important roles in cancer’s genesis, development, metastasis, and response to treatment [59, 60, 61]. The neurobiology of cancer is an emerging discipline spawning new perspectives in oncology. The discovery of multiple pathways related to neural systems that are associated with different cancer types indicates that neural systems may contribute to cancer racial disparities. It also confirms that these neural pathways may indeed play important roles in cancer. There have been few discoveries in previous cancer racial disparity studies pointing to neural pathways as associated genetic factors. Whether they act as drivers will require additional investigation in the future.

## Conflict of interests

The authors declare no conflict of interest.

## Authors contributions

JZ, YL, and QS originally designed the project and wrote this article. PZ and TZ conceptualized the research and revised the manuscript. XP analyzed the data and generated results both in this study and online. ZC performed the Bayesian network modeling. YZ, FM, YL, XZ, and SS collected data and prepared tables and figures. All authors have read and approved the final manuscript.

## Supporting information


**Fig. S1.** Survival plots of XKR9 and CST1.
**Fig. S2.** Heatmap of the selected top 30 differentially expressed genes.
**Fig. S3.** The correlation matrix of the expressions of the genes in Figure 1D.
**Fig. S4.** Location of the SNP, rs17689585, associated with XKR9 gene expression.
**Fig. S5.** The correlation matrix of the expressions of transcription factors that may regulate XKR9.
**Table S1.** Genetic and epigenetic factors reported in previous studies that may be associated with racial disparity of cancer.
**Table S2.** The pathways associated with genes identified through Bayesian network modeling of XKR9.
**Table S3.** Sequence homology‐based prediction of damaging coding SNPs using SIFT.
**Table S3.** Sequence homology‐based prediction of damaging coding SNPs using SIFT.
