# Supplementary material for: Genetic factors associated with cancer racial disparity – an integrative study across twenty‐one cancer types
Source: Mol Oncol. 2020 Sep 24;14(11):2775–86. doi: 10.1002/1878-0261.12799 (PMC7607166; doi:10.1002/1878-0261.12799)
Supplement: Supplementary file 1 — Fig. S1. Survival plots of XKR9 and CST1. Fig. S2. Heatmap of the selected top 30 differentially expressed genes. Fig. S3. The correlation matrix of the expressions of the genes in Figure 1D. Fig. S4. Location of the SNP, rs17689585, associated with XKR9 gene expression. Fig. S5. The correlation matrix of the expressions of transcription factors that may regulate XKR9. Table S1. Genetic and epigenetic factors reported in previous studies that may be associated with racial disparity of cancer. Table S2. The pathways associated with genes identified through Bayesian network modeling of XKR9. Table S3. Sequence homology‐based prediction of damaging coding SNPs using SIFT. Table S3. Sequence homology‐based prediction of damaging coding SNPs using SIFT. [file MOL2-14-2775-s001.docx]

# Genetic factors associated with cancer racial disparity - an integrative study comparing three major races across twenty-one cancer types

**Yan Li, Xiaodong Pang, Zihan Cui, Yidong Zhou, Feng Mao, Yan Lin, Xiaohui Zhang, Songjie Shen, Peixin Zhu, Tingting Zhao, Qiang Sun, Jinfeng Zhang**

**
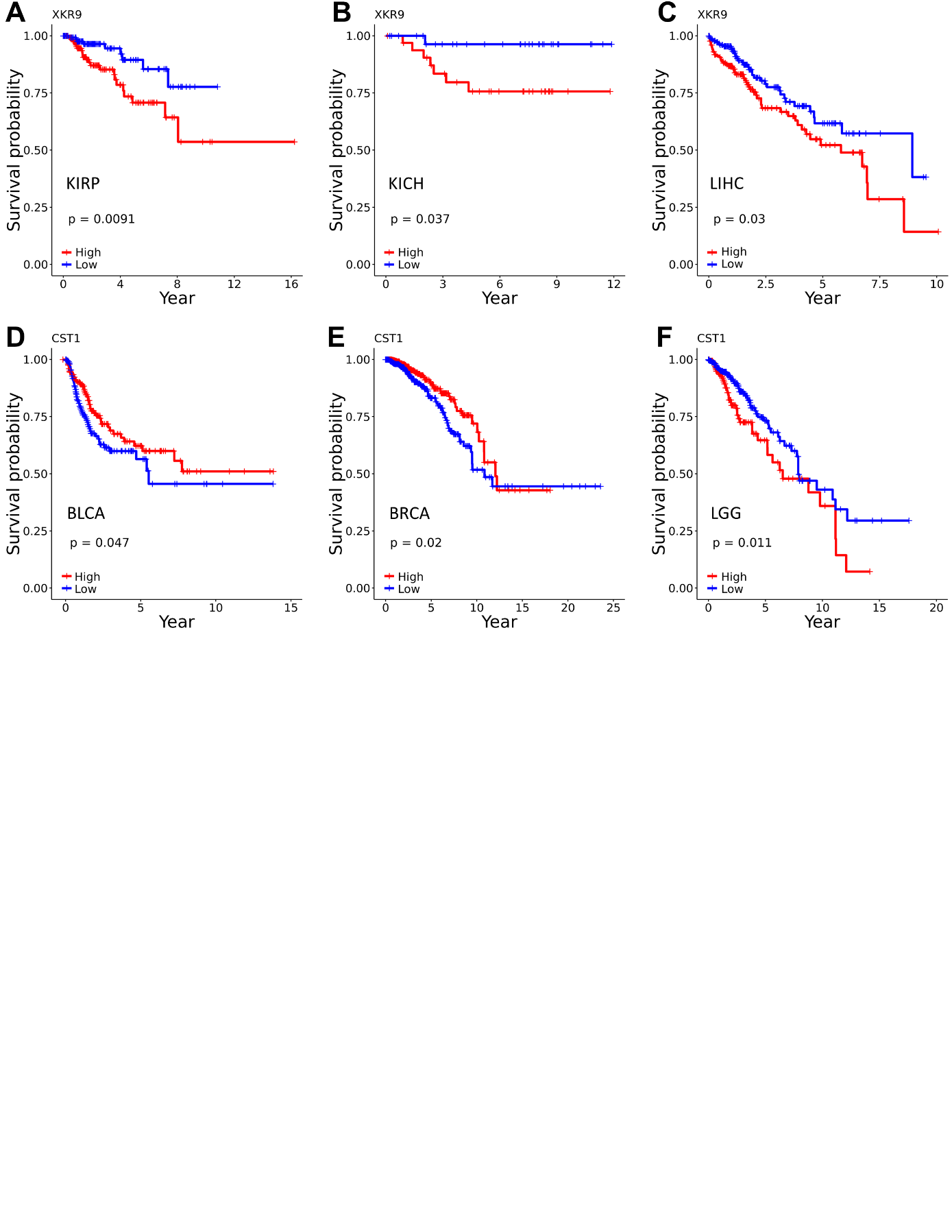
**

**Figure S1.** **Survival plots of XKR9 and CST1.** The median value of XKR9 or CST1 expression was used to separate the population into high and low groups. For XKR9, (A) Kidney renal papillary cell carcinoma (KIRP); (B) Liver hepatocellular carcinoma (LIHC); (C) Kidney chromophobe (KICH). For CST1, (D) Bladder Urothelial Carcinoma (BLCA), (E) Breast invasive carcinoma, (F) Brain Lower Grade Glioma (LGG).

**
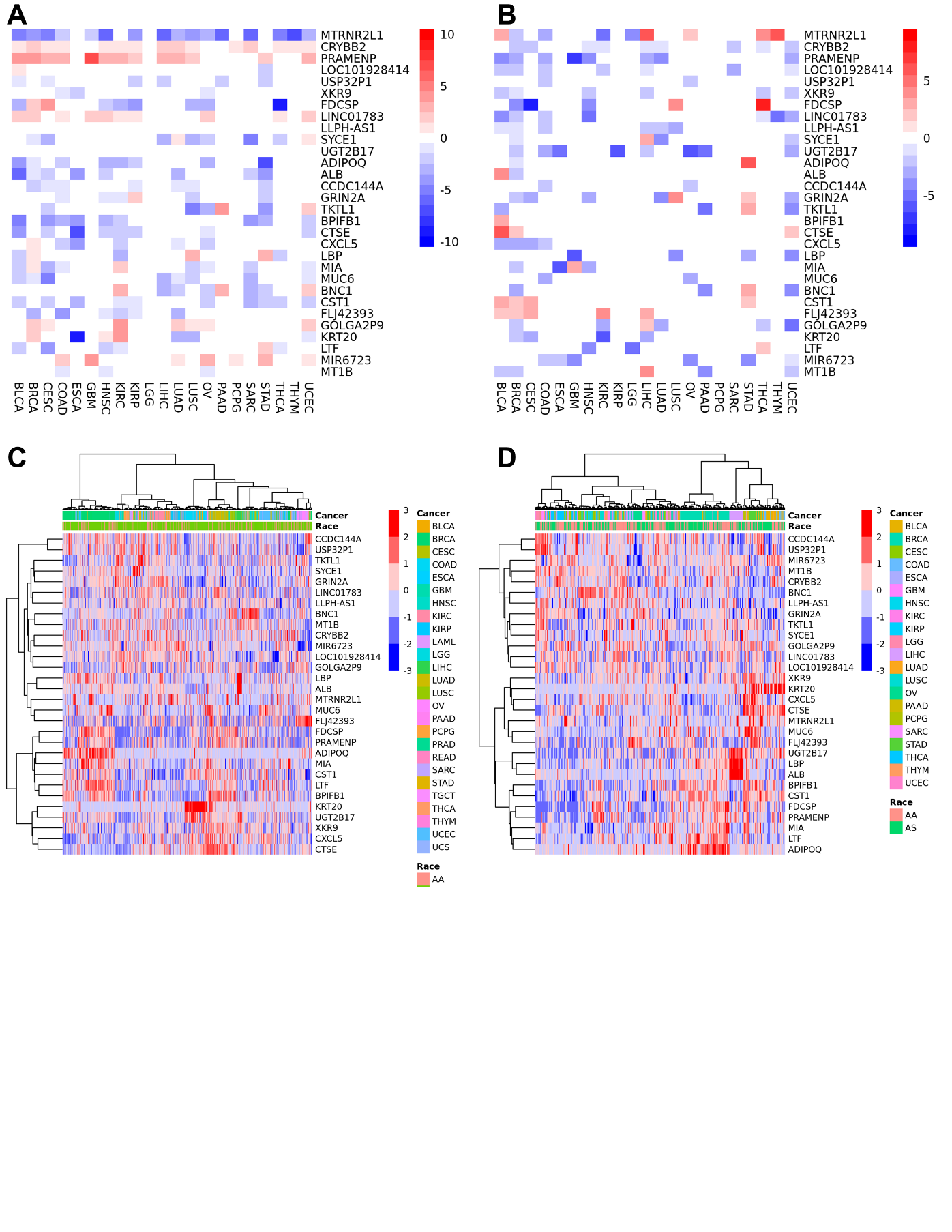
**

**Figure S2. Heatmap of the selected top 30 differentially expressed genes.** (A, B) log2 fold-change for AA vs CA and AS vs AA, respectively. (C, D) gene expression for AA vs CA and AS vs AA, respectively.

**
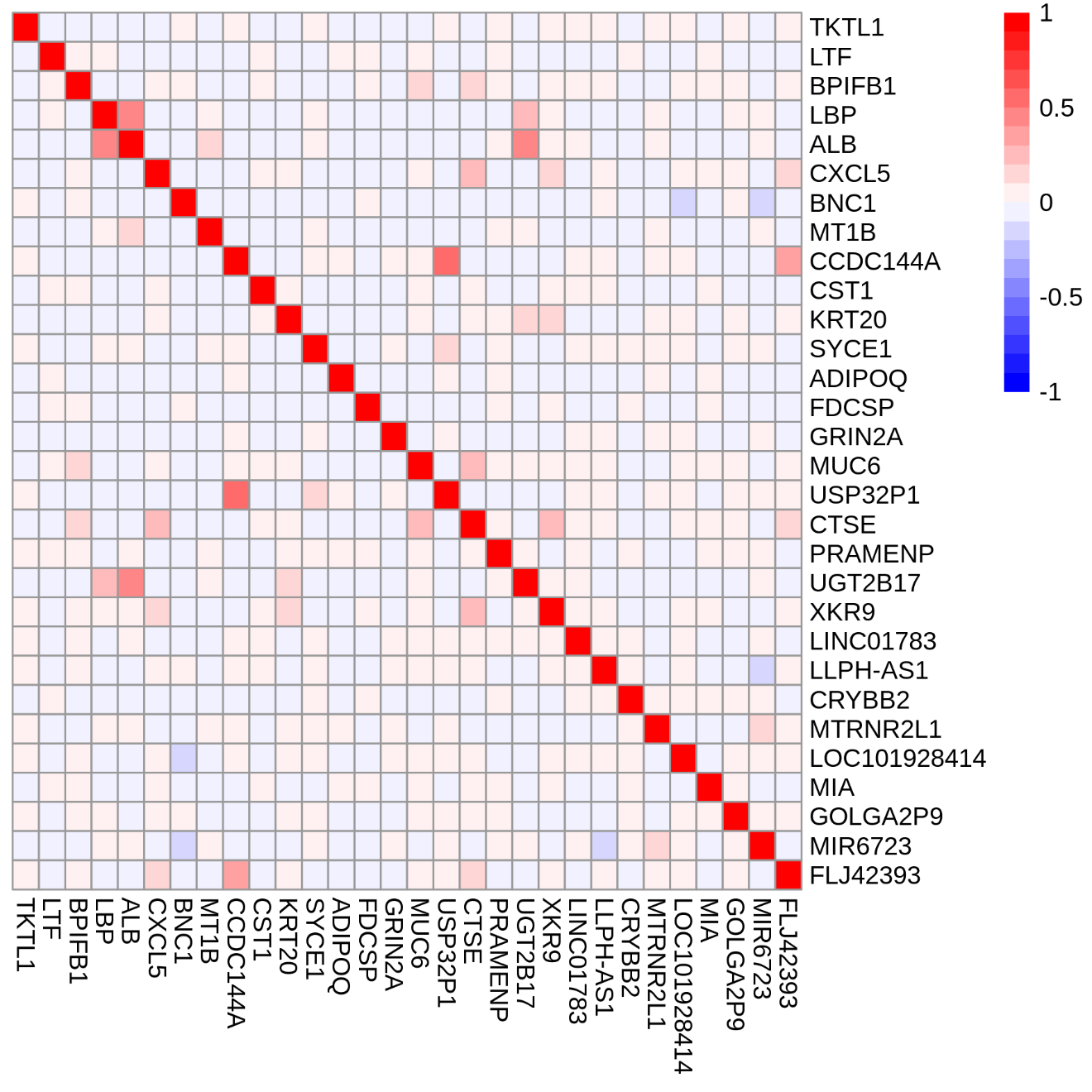
**

**Figure S3. The correlation matrix of the expressions of the genes in Figure 1D.**

**
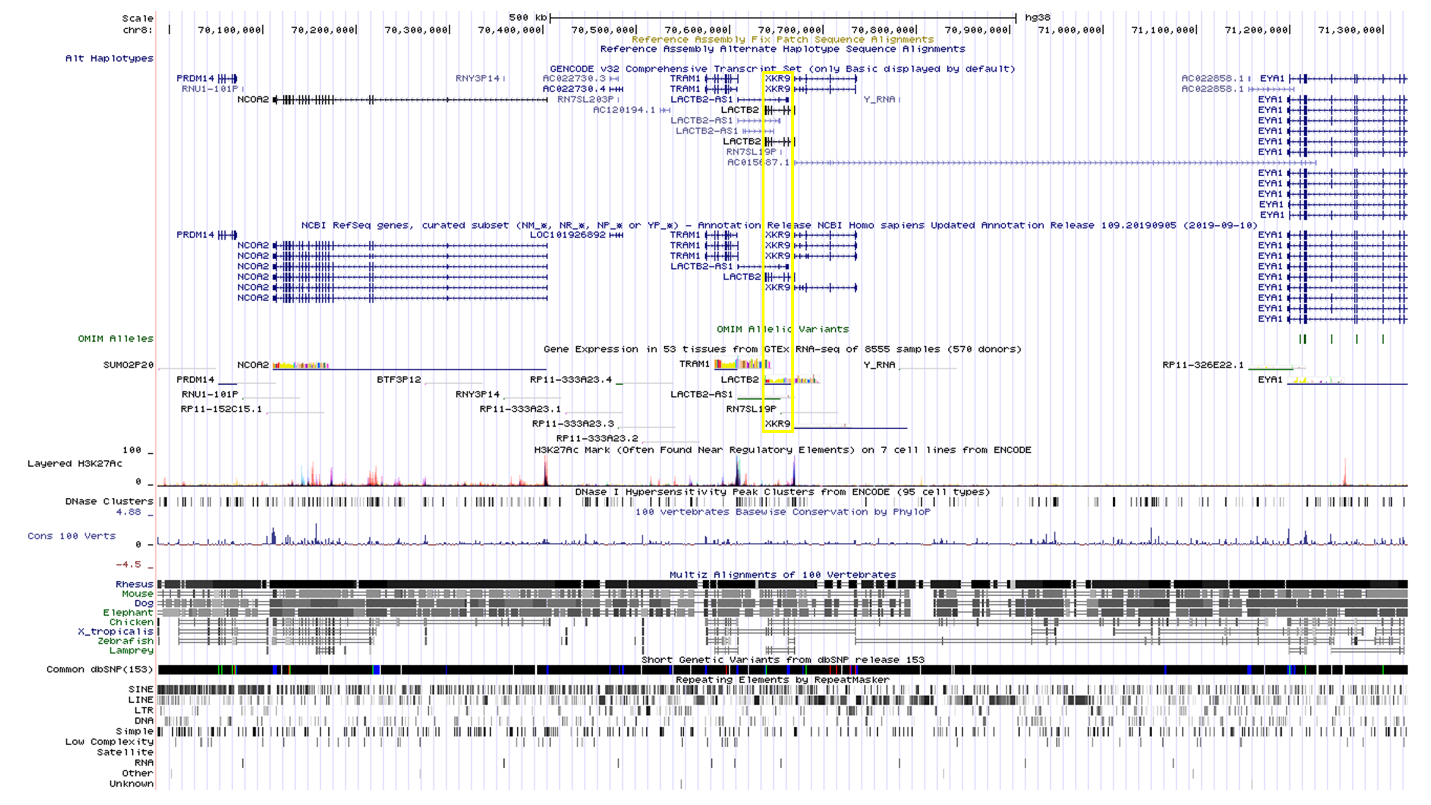
**

**Figure S4. Location of the SNP, rs17689585, associated with XKR9 gene expression.** XKR9 is highlighted in yellow box. Click this [URL](https://genome.ucsc.edu/cgi-bin/hgTracks?db=hg38&lastVirtModeType=default&lastVirtModeExtraState=&virtModeType=default&virtMode=0&nonVirtPosition=&position=chr8%3A69986523%2D71326522&hgsid=843709391_0hddVh194uan98JCKbTkwfRJhSh3) link to see it interactively on UCSC browser.

**
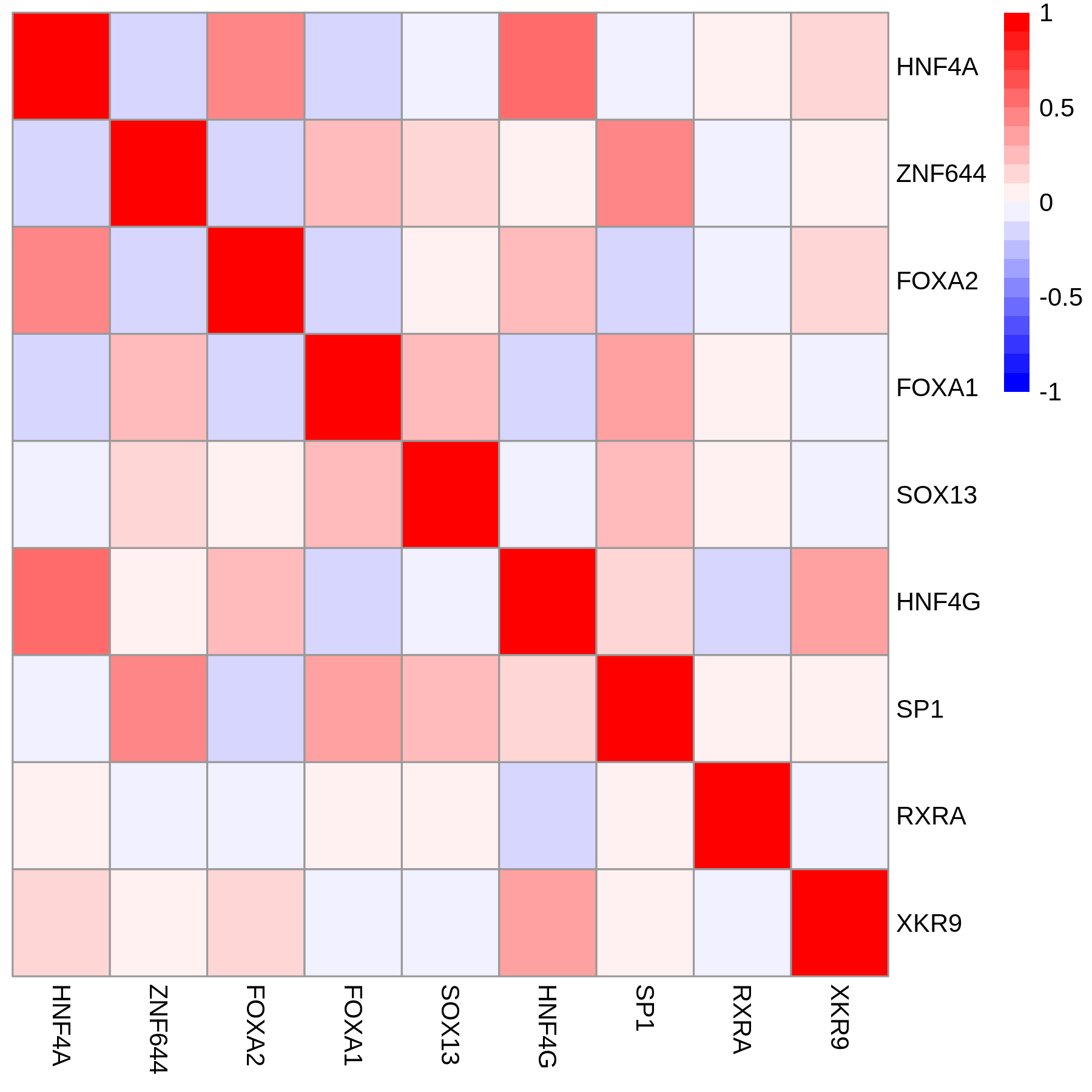
**

**Figure S5. The correlation matrix of the expressions of transcription factors that may regulate XKR9.** We can see that HNF4A, HNF4G and FOXA2 are all highly correlated with XKR9.

**Table S1.** Genetic and epigenetic factors reported in previous studies that may be associated with racial disparity of cancer. AA: African American; CA: Caucasian American; AS: Asian American. Top genetic factors in each study are included in the table.

| Cancer | Genetic/epigenetic factors | races | ref. |
| --- | --- | --- | --- |
| breast | RETN, CRYBB2, TREML4, CXCL10, LOC90784 | AA, CA | [1] |
| breast | CYP4Z1, CYP2A6, UGTs, CYP2D6, ADH | AA, AS, CA | [2] |
| breast | PCSK1, CIDEC, GLYAT, PPARs, CYP1A1 | AS, CA | [3] |
| breast | BRCA1 and BRCA2 germline mutation. | AA, CA | [4] |
| breast | PSPHL, TMPO, AK2, NBN, CRYBB2 | AA, CA | [5] |
| breast | CRYBB2, PSPHL, SOS1, ZFP112, WDR48 | AA, CA | [6] |
| breast | CDKN1B, GATA3, AR, CDKN1A, MUC1 | AA, CA | [7] |
| breast | SNP rs10069690 at TERT-CLPTM1L | AA, CA | [8] |
| breast | SNP rs3104746 in LOC643714 locus | AA, CA | [9] |
| breast | SNPs rs10069690 (TERT) and rs8170 | AA | [10] |
| prostate | 8q24 is a risk locus | AA | [11] |
| prostate | CRYBB2, PSPHL, CTNNB1, C8orf4, AMFR | AA, CA | [12] |
| prostate | SOS1, AMFR, PSPHL, CRYBB2, CTNNB1 | AA, CA | [13] |
| prostate | Allele −8 of the microsatellite DG8S737 | AA, CA | [14] |
| prostate | SNPs rs4430796, rs1859962, rs16901979, rs6983267, rs1447295 (three of them are at 8q24) | AA, CA | [15] |
| prostate | Multiple regions within 8q24 | AA, CA | [16] |
| Colon | SA1 | AA, CA | [17, 18] |
| Colon | AA and CA colon cancers do not have significant differences | AA, CA | [19] |
| Lung | fewer mutations and fewer copy number alterations | AS (compared to CA) | [20] |

**Table S2**. The pathways associated with genes identified through Bayesian network modeling of XKR9.

| **Name** | **Symbol** | **Pathway** |
| --- | --- | --- |
| FOXM1 | FOXM1 | Breast Cancer Regulation by Stathmin1, HOTAIR Regulatory Pathway |
| Cyclin B1 | CCNB1 | Sonic Hedgehog Signaling, GADD45 Signaling, DNA damage-induced 14-3-3σ Signaling, Cell Cycle: G2/M DNA Damage Checkpoint Regulation, Mitotic Roles of Polo-Like Kinase, ATM Signaling, Hereditary Breast Cancer Signaling, Cyclins and Cell Cycle Regulation, Senescence Pathway |
| HLTF | HLTF | Glucocorticoid Receptor Signaling, AMPK Signaling, Role of JAK2 in Hormone-like Cytokine Signaling, RAR Activation, Role of BRCA1 in DNA Damage Response, Hereditary Breast Cancer Signaling |
| TGS1 | TGS1 | PPARα/RXRα Activation |
| NMBR | NMBR | Breast Cancer Regulation by Stathmin1 |

**Table S3.** Sequence homology-based prediction of damaging coding SNPs using SIFT.

| **SNP rsID** | **Reference Allele** | **Variant Allele** | **Substitution** | **Gene Name** | **SIFT Score** | **SIFT Prediction** |
| --- | --- | --- | --- | --- | --- | --- |
| rs6991329 | G | A | A44A | XKR9 | 0.13 | Tolerated |
| rs3810110 | G | A | A82V | SIGLEC12 | 0.278 | Tolerated |
| rs3829658 | G | A | T478M | SIGLEC12 | 0.228 | Tolerated |
| rs3829658 | G | A | T360M | SIGLEC12 | 0.237 | Tolerated |
| rs2009362 | G | A | G350G | SIGLEC12 | 1 | Tolerated |
| rs2009362 | G | A | G232G | SIGLEC12 | 1 | Tolerated |
| rs7406516 | G | A | K351K | TSPAN10 | 1 | Tolerated |
| rs3752135 | T | G | Y376S | SIGLEC12 | 0.601 | Tolerated |
| rs3752135 | T | G | Y494S | SIGLEC12 | 0.777 | Tolerated |
| rs2020870 | A | G | D36G | FMO2 | 0.016 | Deleterious |
| rs6135048 | C | A | G173V | SIRPB2 | 0 | Deleterious |
| rs11854484 | C | T | P22L | SLC28A2 | 0.316 | Tolerated |
| rs35931837 | C | T | G278S | SIGLEC6 | 1 | Tolerated |
| rs35931837 | C | T | G262S | SIGLEC6 | 1 | Tolerated |
| rs35931837 | C | T | G289S | SIGLEC6 | 1 | Tolerated |
| rs35931837 | C | T | G267S | SIGLEC6 | 1 | Tolerated |
| rs35931837 | C | T | G278S | SIGLEC6 | 1 | Tolerated |
| rs35931837 | C | T | G226S | SIGLEC6 | 1 | Tolerated |
|  |  |  |  |  |  |  |

**Prediction of damaging SNPs using SIFT.**

Computational in-silico analysis using SIFT can predict 90% of damaging SNPs. A widely used program called SIFT [21] were used for prediction of damaging SNPs. Of the 291 SNPs identified to significantly (with q-value < 0.5) associated with DEGs for AS vs CA comparison (Figure 3D), two SNPs (rs2020870, rs6135048) were predicted to be deleterious with SIFT score < 0.05 and 8 SNPs being tolerated.

**Data**

**Genomic data from TCGA**. Processed, de-identified, RNA-seq data from The Cancer Genome Atlas (TCGA) were downloaded from the Recount2 database (Collado-Torres L, Nellore A, Kammers K, et al. (2017) Reproducible RNA-seq analysis using recount2. *Nat Biotechnol*. 35(4):319-321. doi:10.1038/nbt.3838). When studying individual cancers, only cancers with at least five patients from each racial group were selected for study, which resulted in the selection of the following 21 cancer types: BLCA, BRCA, CESC, COAD, ESCA, GBM, HNSC, KIRC, KIRP, LGG, LIHC, LUAD, LUSC, OV, PAAD, PCPG, SARC, STAD, THCA, THYM, and UCEC. In total, there were 834 African American (AA) samples, 602 Asian American (AS) samples, and 6353 Caucasian American (CA) samples. Hispanics were not considered as a separate race/ethnicity group because previous studies did not separate Hispanics from Caucasian population, especially those using TCGA data [1]. When studying the differentially expressed genes in all cancers combined, we matched each patient sample from the AA and AS groups with four (or very close to four) patient samples of the same cancer type from the CA group. When comparing AA samples to AS samples, the ratio of the patient samples from each race is 1:1 for each cancer type. In total, 468 AS samples from 23 cancer types were matched with 1867 CA samples; 847 AA samples were matched to 3383 CA samples for 26 cancer types; and 289 AA samples were matched with 289 AS samples from 21 cancer types. Data on select clinical variables (e.g., disease status, cancer type) were also obtained. Custom Python scripts (The Python Software Foundation, http://www.python.org) were used to integrate the data for analysis.

**SNP-gene expression associations from GTEx.** Tissue Expression Quantitative Trait Loci (eQTL) data were downloaded from the Genotype-Tissue Expression (GTEx) data portal (<https://gtexportal.org/>) for the differentially expressed genes identified from the various differential gene expression analyses.

**1000 Genomes Project data.** Allele frequency data for the identified SNPs in the three racial groups were extracted from data downloaded from the 1000 Genomes NCBI FTP site (<ftp://ftp-trace.ncbi.nih.gov/1000genomes/ftp>).

**Kallikrein (KLK) genes**

The human kallikrein (KLK) gene family has 15 members all located on chromosome 19q13.4. KLK3, or prostate-specific antigen (PSA), has been widely used in prostate cancer screening. Kallikreins are known to play a role in several physiological processes including extracellular matrix (ECM) remodeling, differentiation, apoptosis, angiogenesis, cellular proliferation, digestive system enzyme activation and coagulation-fibrinolysis. The potential role of KLKs as cancer biomarkers for diagnosis and prognosis have been well recognized [22, 23]. KLKs are down-regulated in several cancers in AS cancer patients compared to their CA and AA counterparts. With this in mind, when a KLK member gene is developed as a biomarker in western countries, it should be tested in Asian countries before being adopted clinically. This is necessary since many Asian cancer patients have low expression levels of KLK genes, and therefore members of this gene family may not serve as good biomarkers in that population. Even they are used as biomarkers, their behaviors are likely different and different models should be built for patient populations in Asian countries.

**Immune response regulators SIGLEC12 and SIGLEC14**

In Table 1, SIGLEC14 and SIGLEC12 were identified as differentially expressed genes but with different patterns of differential expression. SIGLECs were found to be regulators of immune cell function and have garnered much interest in recent years [24][. Activating Siglecs are frequently paired with inhibitory counterparts. SIGLEC14 is likely an activating Siglec](#_ENREF_56) [, while SIGLEC12 likely plays an inhibitory role](#_ENREF_56) [25][. Thus, the expression patterns of SIGLEC14 and SIGLEC12 are consistent with their roles, and together their differential expression may explain some of the observed racial disparity among AS, CA and AA cancers.](#_ENREF_57)

Finally, we conclude with a discussion of the significance of studying genetic factors for cancer racial disparities. We believe that a better understanding of the genetic factors associated with cancer racial disparities will: (1) help researchers design effective approaches to reduce/eliminate cancer racial disparities by investing resources in the right areas; (2) help researchers understand cancer biology in general (the discovery of XKR9 as a prognostic biomarker is an example of such potential benefit); (3) help researchers better understand cancer heterogeneity and design better personalized treatments; (4) help researchers focus drug development on neglected targets affecting under-represented populations; and (5) enable drug development efforts to design better clinical trials when the drug target or biomarker is associated with racial disparity.

**References**

1. Stewart P A, Luks J, Roycik M D, Sang Q X & Zhang J (2013) Differentially expressed transcripts and dysregulated signaling pathways and networks in African American breast cancer. PLoS One 8, e82460.

2. Li Y, Steppi A, Zhou Y, Mao F, Miller P C, He M M, Zhao T, Sun Q & Zhang J (2017) Tumoral expression of drug and xenobiotic metabolizing enzymes in breast cancer patients of different ethnicities with implications to personalized medicine. Sci Rep 7, 4747.

3. Shi Y, Steppi A, Cao Y, Wang J, He M M, Li L & Zhang J (2017) Integrative Comparison of mRNA Expression Patterns in Breast Cancers from Caucasian and Asian Americans with Implications for Precision Medicine. Cancer Res 77, 423-433.

4. Nanda R, Schumm L P, Cummings S, Fackenthal J D, Sveen L, Ademuyiwa F, Cobleigh M, Esserman L, Lindor N M, Neuhausen S L & Olopade O I (2005) Genetic testing in an ethnically diverse cohort of high-risk women: a comparative analysis of BRCA1 and BRCA2 mutations in American families of European and African ancestry. JAMA 294, 1925-33.

5. Martin D N, Boersma B J, Yi M, Reimers M, Howe T M, Yfantis H G, Tsai Y C, Williams E H, Lee D H & Stephens R M (2009) Differences in the tumor microenvironment between African-American and European-American breast cancer patients. PloS one 4, e4531.

6. Field L A, Love B, Deyarmin B, Hooke J A, Shriver C D & Ellsworth R E (2012) Identification of differentially expressed genes in breast tumors from African American compared with Caucasian women. Cancer 118, 1334-44.

7. Grunda J M, Steg A D, He Q, Steciuk M R, Byan-Parker S, Johnson M R & Grizzle W E (2012) Differential expression of breast cancer-associated genes between stage- and age-matched tumor specimens from African- and Caucasian-American Women diagnosed with breast cancer. BMC research notes 5, 248.

8. Haiman C A, Chen G K, Vachon C M, Canzian F, Dunning A, Millikan R C, Wang X, Ademuyiwa F, Ahmed S, Ambrosone C B, Baglietto L, Balleine R, Bandera E V, Beckmann M W, Berg C D, Bernstein L, Blomqvist C, Blot W J, Brauch H, Buring J E, Carey L A, Carpenter J E, Chang-Claude J, Chanock S J, Chasman D I, Clarke C L, Cox A, Cross S S, Deming S L, Diasio R B, Dimopoulos A M, Driver W R, Dunnebier T, Durcan L, Eccles D, Edlund C K, Ekici A B, Fasching P A, Feigelson H S, Flesch-Janys D, Fostira F, Forsti A, Fountzilas G, Gerty S M, Giles G G, Godwin A K, Goodfellow P, Graham N, Greco D, Hamann U, Hankinson S E, Hartmann A, Hein R, Heinz J, Holbrook A, Hoover R N, Hu J J, Hunter D J, Ingles S A, Irwanto A, Ivanovich J, John E M, Johnson N, Jukkola-Vuorinen A, Kaaks R, Ko Y D, Kolonel L N, Konstantopoulou I, Kosma V M, Kulkarni S, Lambrechts D, Lee A M, Marchand L L, Lesnick T, Liu J, Lindstrom S, Mannermaa A, Margolin S, Martin N G, Miron P, Montgomery G W, Nevanlinna H, Nickels S, Nyante S, Olswold C, Palmer J, Pathak H, Pectasides D, Perou C M, Peto J, Pharoah P D, Pooler L C, Press M F, Pylkas K, Rebbeck T R, Rodriguez-Gil J L, Rosenberg L, Ross E, Rudiger T, Silva Idos S, Sawyer E, Schmidt M K, Schulz-Wendtland R, Schumacher F, Severi G, Sheng X, Signorello L B, Sinn H P, Stevens K N, Southey M C, Tapper W J, Tomlinson I, Hogervorst F B, Wauters E, Weaver J, Wildiers H, Winqvist R, Van Den Berg D, Wan P, Xia L Y, Yannoukakos D, Zheng W, Ziegler R G, Siddiq A, Slager S L, Stram D O, Easton D, Kraft P, Henderson B E & Couch F J (2011) A common variant at the TERT-CLPTM1L locus is associated with estrogen receptor-negative breast cancer. Nat Genet 43, 1210-4.

9. Ruiz-Narvaez E A, Rosenberg L, Cozier Y C, Cupples L A, Adams-Campbell L L & Palmer J R (2010) Polymorphisms in the TOX3/LOC643714 locus and risk of breast cancer in African-American women. Cancer Epidemiol Biomarkers Prev 19, 1320-7.

10. Palmer J R, Ruiz-Narvaez E A, Rotimi C N, Cupples L A, Cozier Y C, Adams-Campbell L L & Rosenberg L (2013) Genetic susceptibility loci for subtypes of breast cancer in an African American population. Cancer Epidemiol Biomarkers Prev 22, 127-34.

11. Freedman M L, Haiman C A, Patterson N, McDonald G J, Tandon A, Waliszewska A, Penney K, Steen R G, Ardlie K, John E M, Oakley-Girvan I, Whittemore A S, Cooney K A, Ingles S A, Altshuler D, Henderson B E & Reich D (2006) Admixture mapping identifies 8q24 as a prostate cancer risk locus in African-American men. Proc Natl Acad Sci U S A 103, 14068-73.

12. Wallace T A, Prueitt R L, Yi M, Howe T M, Gillespie J W, Yfantis H G, Stephens R M, Caporaso N E, Loffredo C A & Ambs S (2008) Tumor immunobiological differences in prostate cancer between African-American and European-American men. Cancer Res 68, 927-36.

13. Timofeeva O A, Zhang X, Ressom H W, Varghese R S, Kallakury B V, Wang K, Ji Y, Cheema A, Jung M, Brown M L, Rhim J S & Dritschilo A (2009) Enhanced expression of SOS1 is detected in prostate cancer epithelial cells from African-American men. Int J Oncol 35, 751-60.

14. Amundadottir L T, Sulem P, Gudmundsson J, Helgason A, Baker A, Agnarsson B A, Sigurdsson A, Benediktsdottir K R, Cazier J B, Sainz J, Jakobsdottir M, Kostic J, Magnusdottir D N, Ghosh S, Agnarsson K, Birgisdottir B, Le Roux L, Olafsdottir A, Blondal T, Andresdottir M, Gretarsdottir O S, Bergthorsson J T, Gudbjartsson D, Gylfason A, Thorleifsson G, Manolescu A, Kristjansson K, Geirsson G, Isaksson H, Douglas J, Johansson J E, Balter K, Wiklund F, Montie J E, Yu X, Suarez B K, Ober C, Cooney K A, Gronberg H, Catalona W J, Einarsson G V, Barkardottir R B, Gulcher J R, Kong A, Thorsteinsdottir U & Stefansson K (2006) A common variant associated with prostate cancer in European and African populations. Nat Genet 38, 652-8.

15. Zheng S L, Sun J, Wiklund F, Smith S, Stattin P, Li G, Adami H O, Hsu F C, Zhu Y, Balter K, Kader A K, Turner A R, Liu W, Bleecker E R, Meyers D A, Duggan D, Carpten J D, Chang B L, Isaacs W B, Xu J & Gronberg H (2008) Cumulative association of five genetic variants with prostate cancer. N Engl J Med 358, 910-9.

16. Haiman C A, Patterson N, Freedman M L, Myers S R, Pike M C, Waliszewska A, Neubauer J, Tandon A, Schirmer C, McDonald G J, Greenway S C, Stram D O, Le Marchand L, Kolonel L N, Frasco M, Wong D, Pooler L C, Ardlie K, Oakley-Girvan I, Whittemore A S, Cooney K A, John E M, Ingles S A, Altshuler D, Henderson B E & Reich D (2007) Multiple regions within 8q24 independently affect risk for prostate cancer. Nat Genet 39, 638-44.

17. Wali R K, Momi N, Dela Cruz M, Calderwood A H, Stypula-Cyrus Y, Almassalha L, Chhaparia A, Weber C R, Radosevich A, Tiwari A K, Latif B, Backman V & Roy H K (2016) Higher Order Chromatin Modulator Cohesin SA1 Is an Early Biomarker for Colon Carcinogenesis: Race-Specific Implications. Cancer Prev Res (Phila) 9, 844-854.

18. Datta S, Sherva R M, De La Cruz M, Long M T, Roy P, Backman V, Chowdhury S & Roy H K (2018) Single Nucleotide Polymorphism Facilitated Down-Regulation of the Cohesin Stromal Antigen-1: Implications for Colorectal Cancer Racial Disparities. Neoplasia 20, 289-294.

19. Govindarajan R, Posey J, Chao C Y, Lu R, Jadhav T, Javed A Y, Javed A, Mahmoud F A, Osarogiagbon R U & Manne U (2016) A comparison of 12-gene colon cancer assay gene expression in African American and Caucasian patients with stage II colon cancer. BMC Cancer 16, 368.

20. Chen J, Yang H, Teo A S M, Amer L B, Sherbaf F G, Tan C Q, Alvarez J J S, Lu B, Lim J Q, Takano A, Nahar R, Lee Y Y, Phua C Z J, Chua K P, Suteja L, Chen P J, Chang M M, Koh T P T, Ong B H, Anantham D, Hsu A A L, Gogna A, Too C W, Aung Z W, Lee Y F, Wang L, Lim T K H, Wilm A, Choi P S, Ng P Y, Toh C K, Lim W T, Ma S, Lim B, Liu J, Tam W L, Skanderup A J, Yeong J P S, Tan E H, Creasy C L, Tan D S W, Hillmer A M & Zhai W (2020) Genomic landscape of lung adenocarcinoma in East Asians. Nat Genet 52, 177-186.

21. Vaser R, Adusumalli S, Leng S N, Sikic M & Ng P C (2016) SIFT missense predictions for genomes. Nat Protoc 11, 1-9.

22. Diamandis E P & Yousef G M (2002) Human tissue kallikreins: a family of new cancer biomarkers. Clin Chem 48, 1198-205.

23. Tailor P D, Kodeboyina S K, Bai S, Patel N, Sharma S, Ratnani A, Copland J A, She J X & Sharma A (2018) Diagnostic and prognostic biomarker potential of kallikrein family genes in different cancer types. Oncotarget 9, 17876-17888.

24. Fraschilla I & Pillai S (2017) Viewing Siglecs through the lens of tumor immunology. Immunol Rev 276, 178-191.

25. Flores R, Zhang P, Wu W, Wang X, Ye P, Zheng P & Liu Y (2019) Siglec genes confer resistance to systemic lupus erythematosus in humans and mice. Cell Mol Immunol 16, 154-164.
